# Barriers to cervical cancer screening in Africa: a systematic review

**DOI:** 10.1186/s12889-024-17842-1

**Published:** 2024-02-20

**Authors:** Fennie Mantula, Yoesrie Toefy, Vikash Sewram

**Affiliations:** 1 African Cancer Institute, Stellenbosch University, P.O Box 241, Cape Town, 8000 South Africa; 2 Division of Health Systems and Public Health, Department of Global Health, Faculty of Medicine and Health Sciences, Stellenbosch University, P.O Box 241, Cape Town, 8000 South Africa; 3 Department of Nursing and Midwifery, Faculty of Medicine, National University of Science and Technology, P.O. Box A.C. 939, Ascot, Bulawayo, Zimbabwe

**Keywords:** Cervical cancer, Cervical cancer screening, Barriers, Systematic review, Africa

## Abstract

**Introduction:**

Africa has one of the highest burdens of cervical cancer in the world. The unacceptably high incidence and mortality rates could be reduced through implementing a comprehensive approach to its prevention and control that includes screening, which however, is low in most low-and-middle-income countries. Hence, this systematic review aims at exploring factors that prevent women from utilising cervical cancer screening services in the region.

**Methods:**

A mixed method systematic review was conducted. A search was performed on PubMed (Medline), EMBASE, CINAHL (EBSCOHOST) and Scopus databases for articles published until May 2019 without time, language or study design limits. Two reviewers critically appraised the included studies independently using the standard quality assessment criteria for evaluating primary research papers. Results of the quantitative and mixed methods studies were transformed into qualitative data and synthesised using thematic analysis.

**Results:**

From a potential 2 365 studies, 24 from 11 countries met the eligibility criteria and were selected; eight qualitative, 13 quantitative, and three that used the mixed-method approach. The primary barriers were identified as poor access to screening services, lack of awareness and knowledge on cervical cancer and screening, and socio-cultural influences. Service providers perceived lack of skills, screening equipment and supplies, and staff shortages as the major barriers to the provision of screening services.

**Conclusion:**

Barriers to cervical cancer screening in Africa are multifaceted and require a holistic approach that will address them concurrently at the health system, individual, interpersonal, community and structural levels. Political will complimented by stakeholder involvement is required in the development and implementation of strategies that will ensure acceptability, availability, accessibility, and affordability of screening to minimise barriers in accessing the service.

**Supplementary Information:**

The online version contains supplementary material available at 10.1186/s12889-024-17842-1.

## Introduction

Cervical cancer is the fourth most common cancer among women worldwide with an estimated 604,127 new cases and 341,831 deaths reported in 2020 [1], up from 528,000 new cases and 266,000 deaths reported in 2012 [2]. The bulk of the global burden rests with Africa, Latin America, the Caribbean and Asia where approximately 90% of deaths occur [3]. With an estimated population of 372.2 million women aged 15 years and older who are at risk of developing cervical cancer in Africa, 119, 284 women are diagnosed with cervical cancer while 81,687 die from the disease every year [4]. Compared to other regions in the world, Africa has higher cervical cancer incidence and mortality rates [1, 3, 5]. Cervical cancer screening can reduce the incidence of the disease by 70–80% if targeted appropriately [6, 7]. However, in many parts of Africa, the disease is often not identified until it reaches advanced stages that are associated with poor outcomes [8]. This has been attributed to lack of comprehensive cervical cancer screening programmes in most countries [5]. Cervical cancer is the most preventable cancer due to its slow progression and early identifiable precancerous lesions which can be treated before they progress to cancer [9] hence, women need not die from cervical cancer.

Primary studies have been conducted over the past decades to identify barriers to the uptake of cervical cancer screening in various African countries. Although limited, systematic reviews have also been done to look into challenges which women encounter in accessing cervical cancer screening services in Sub-Saharan Africa [10, 11]. Despite the recommendations that have been made for overcoming the existing barriers, evidence suggests that cervical cancer incidence rates continue to increase in Africa while declining in many developed countries [1]. A richer understanding of the reasons for the underutilisation of cervical cancer screening programmes in Africa requires further exploration. This review therefore aimed at identifying the unique contextual circumstances that prevent women from accessing cervical cancer screening in many parts of Africa. Guided by the Socio-ecological framework adopted from Kaufman and colleagues [12], our systematic review extends the knowledge already available from earlier conducted studies. Findings should guide restructuring of cervical cancer screening policies and guidelines for implementation of proactive context specific interventions that should address the structural, health system, societal, socio-economic and cultural factors at a broader level to overcome screening barriers. This could improve the uptake of screening and subsequently reduce the high cervical cancer morbidity and mortality rates in Africa. Gaps for future research will also be identified.

## Methods

This study was conducted in accordance with the Preferred Reporting Items for Systematic Reviews and Meta-analysis (PRISMA) guidelines [13].

### Search strategy

We subjectively and iteratively developed a comprehensive set of search terms. In the first instance, we checked PubMed (Medline) to identify controlled vocabulary Medical Subject Headings (MeSH) terms related to cervical cancer, and additionally identified key text words based on our knowledge of the field. This yielded three key concepts; cervical cancer, screening (irrespective of screening method), and Africa. The term ‘barrier’ was not used because the concept can be described in many different ways, and we did not want to risk missing some relevant papers. Medline search terms for other electronic databases were modified to conform to their search functions. PubMed (Medline), Embase (OVID), CINAHL (EBSCOHOST) and Scopus electronic bibliographic databases were searched for articles published until May 2019 without language and study design limits. The "related citations" search key in PubMed was further used to identify similar papers. Reference lists of potentially relevant articles were checked manually for additional citations. A detailed search strategy with terminology specific to each database is included (Supplementary File 1).

### Study selection

This systematic review included studies on individual, interpersonal, community, health system and structural factors that prevented women from cervical cancer screening attendance in most African countries. The selection criteria were based on original quantitative and qualitative studies that reported barriers from the perspectives of women and health providers. Studies on women with a confirmed cervical cancer diagnosis were not included in the systematic review. Grey literature and conference abstracts without full articles were also excluded. Although these could have been useful sources containing relatively new information on the research area, it is generally premised that non-peer-reviewed articles are less scientifically rigorous than those that are peer reviewed and published [14].

Our systematic review is grounded on the socio-ecological framework by Kaufman and colleagues which describes the interplay between multiple levels of influence on individual behaviour for the promotion of health [12]. The model suggests that a health outcome is determined by individual, interpersonal, institutional, community and public policy factors [15]. In this study “Barrier” refers to any factor that prevents women from accessing cervical cancer screening from any level of the socio-ecological framework and classified into five areas as follows:

*Individual level barriers*: These are factors at the micro-level that include personal perceptions, knowledge, beliefs and emotions.

*Interpersonal barriers*: These arise from influences from spouse, family and other social networks.

*Community level barriers*: These are a result of influences at higher levels which include traditional and cultural norms, religious beliefs and stigma.

*Health system related barriers*: These are factors within the health system that relate to resources and service delivery.

*Structural barriers*: These are factors related to policy issues and other macro-contextual factors that affect a woman’s health seeking behaviours directly.

**Fig. 1:**
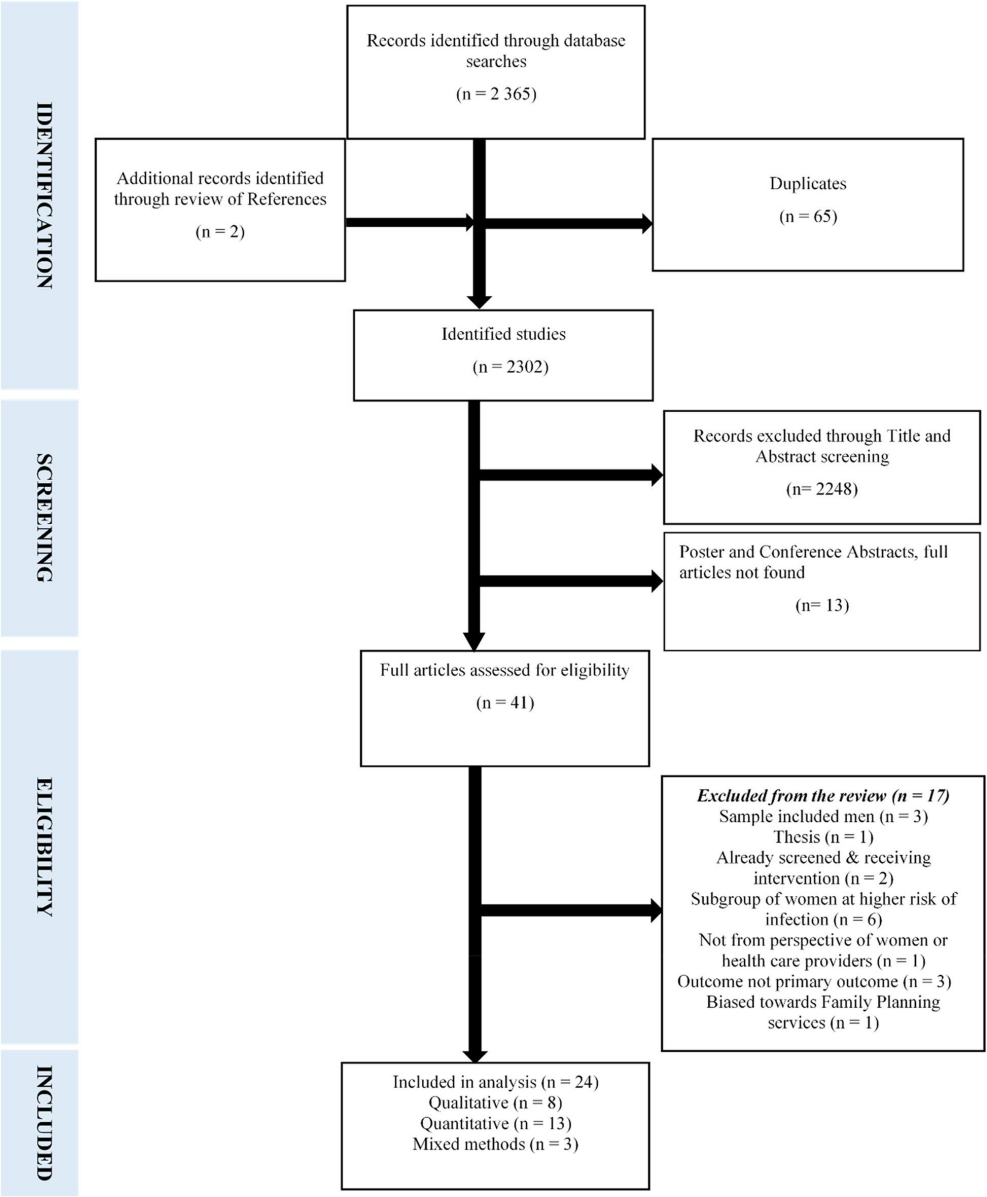
Prisma flow diagram of included studies

Mendeley reference manager was used to save and view titles and abstracts of all articles retrieved from the electronic databases, and to detect duplicates. Two independent reviewers (FM and VS) screened the 2 365 titles and abstracts of studies obtained through database searches. Two additional articles were identified from references after reading the full text articles (*n* = 2 367). Screening of articles excluded duplicates (*n* = 65), studies not relevant to the title (*n* = 2 248), and abstracts of poster and conference presentations whose full articles were not obtained (*n* = 13). The remaining 41 articles were reviewed in full text with 17 studies further eliminated for not meeting the inclusion criteria. The screening process resulted in the selection of 24 articles which met the eligibility criteria. Disagreements on inclusion of certain articles were resolved through discussion to reach a consensus [16]. The selection process is shown in Fig. 1.

### Assessment of methodological quality

The quality of each study was evaluated by two independent reviewers (FM and VS) using the standard quality assessment criteria for evaluating primary research papers adapted from Kmet and colleagues [17]. A checklist specific to each research method required the reviewer to select either; “yes” or “no” to questions focusing on the methodological aspects of each article. This tool was appropriate for assessing the quality of the overall body of evidence given in the heterogeneous literature and helped to gauge the quality of each individual study against set standards. Qualitative studies were evaluated using the following criteria: question or objective clearly described, study design evident and appropriate, context for the study clear, connection to a theoretical framework or wider body of knowledge, sampling strategy described, relevant and justified, data collection methods clearly described and systematic, data analysis clearly described and systematic, and conclusion supported by results [17]. Quantitative studies were assessed for the following aspects: question or objective sufficiently described, study design evident and appropriate, method of subject selection described and appropriate, subject characteristics sufficiently described, sample size appropriate, analytic methods described, justified and appropriate, results reported in sufficient detail, and conclusions supported by the results [17]. The quality of studies which used the mixed methods approach was rated under the dominant method that was discussed first in that particular study.

To further determine the overall risk of bias and the quality of evidence, each reviewed article was given a quality of low, medium or high to inform the decision making. Each quality component was rated 0 to 2 based on the reviewer’s subjective assessment, with a possible least score of 0, and a maximum score of 16. A sum score of the quality components gave the overall quality rating of each article. A score of 0–8 was rated as low, 9–12 as medium and 13–16 as high. For a study to be included, it had to attain a minimum rating of medium. All the included studies fulfilled this requirement. The quality assessment for the qualitative studies is presented in Table 1.

**Table 1:** Quality assessment of qualitative studies

Article	Standard quality assessment criteria for evaluating primary research papers (Kmet et al., 2004)								
	**Question or objectives clearly described**	**Study design evident and appropriate**	**Context of study clear**	**Connection to a theoretical framework or wider body of knowledge**	**Sampling strategy described, relevant and justified**	**Data collection methods clearly described and systematic**	**Data analysis clearly described and systematic**	**Conclusion supported by results**	**Total score / Quality rating**
**Ndikom et al. (2012)** [18]	2	1	2	2	2	2	2	2	15 High
**Mookeng et al. (2010)** [19]	2	2	2	2	2	2	2	2	16 High
**Munthali et al. (2015)** [20]	2	1	2	2	1	1	2	2	13 High
**Oketch et al. (2019)** [21]	2	2	2	2	2	2	2	2	16 High
**Mwaka et al. (2013)** [22]	2	1	2	2	2	2	2	2	15 High
**Ndejjo et al. (2017)** [23]	2	2	2	2	2	2	2	2	16 High
**Modibbo et al. (2016)** [24]	2	1	2	2	2	2	2	2	15 High
**Fort et al. (2011)** [25]	2	1	2	2	2	2	1	2	14 High
**Mangoma et al. (2006)** [26]	2	2	2	2	2	2	2	2	16 High
**Ngugi et al. (2011)** [27]	2	1	2	1	2	2	1	2	13 High

Table 2 presents the quality assessment for the quantitative studies.

**Table 2:** Quality assessment of quantitative studies

Article	Standard quality assessment criteria for evaluating primary research papers (Kmet et al., 2004)								
	**Question or objectives clearly described**	**Study design evident and appropriate**	**Method of subject selection described and appropriate**	**Subject characteristics sufficiently described**	**Sample size appropriate**	**Analytic methods described, justified and appropriate**	**Results described in sufficient detail**	**Conclusions supported by results**	**Total Score**
**Nwankwo et al. (2011)** [28]	2	2	1	2	0	1	2	2	12 Medium
**Compaore et al. (2016)** [29]	2	2	1	2	1	2	2	2	14 High
**Tarwireyi (2005)** [30]	2	2	2	2	0	1	2	2	13 High
**Kress et al. (2015)** [31]	2	1	2	2	1	2	2	2	14 High
**Abiodun et al. (2013)** [32]	2	2	2	0	2	1	1	2	12 Medium
**Okunowo et al. (2018)** [33]	2	2	2	2	2	2	2	2	16 High
**Perng et al. (2013)** [34]	2	1	1	1	1	2	1	1	10 Medium
**Ebu et al. (2015)** [35]	2	2	2	2	2	2	2	2	16 High
**Rosser et al. (2015)** [36]	2	1	2	2	2	2	2	2	15 High
**Chigbu et al. (2011)** [37]	2	2	2	2	1	2	2	2	15 High
**Titiloye et al. (2017)** [38]	2	2	2	2	2	2	2	2	16 High
**Ibekwe et al. (2011)** [39]	2	2	2	2	2	2	2	2	16 High
**Abiodun et al. (2013)** [40]	2	2	2	2	2	1	2	2	15 High
**Getachew et al. (2019)** [41]	2	2	2	2	2	2	2	2	16 High

### Data extraction and synthesis

A data extraction sheet was developed using the following predetermined data fields: first author, country and year of publication, title, research and data collection methods, sampling technique and sample size, and barriers identified for cervical cancer screening. One reviewer extracted the data (FM) while the second (VS) cross checked the extracted data for accuracy. Informed by the variation in the research methodologies between included studies and the multifaceted dimensions of screening barriers given, results of the quantitative and mixed methods studies were transformed into qualitative data and synthesised using thematic analysis. [16]. Data were summarised in descriptive form. A profile of all the studies included in the review highlighting the major screening barriers identified is given in Table 3.

**Table 3:** Profile of included studies showing key findings

Author, year, country	Title	Research method and data collection	Sampling technique Sample size	Major barriers identified
**Ndikom CM & Ofi BA., 2012, Nigeria **[18]	Awareness, perception and factors affecting utilization of cervical cancer screening services in Ibadan, Nigeria: A qualitative study	**Research method:** Qualitative **Data collection:** Focus Group Discussions (FGDs)	Purposive 8 FGDs (n = 82)	Lack of awareness of cervical cancer and facilities for screening Low risk perception Illiteracy (belief that services are for rich people) Financial constraints Fear of having a positive result Attitude of indifference to their health Having many contending issues (too busy) Screening services not easily accessible Poor information dissemination by health workers
**Mookeng, M J et al., 2010, South Africa **[19]	Barriers to cervical cancer screening within private medical practitioners in Soshanguve, South Africa	**Research method:** Qualitative **Data collection:** Interviews Field notes	Purposive n = 6	Age of medical practitioner vs. age of woman Gender of medical practitioner Few opportunities for medical practitioners to conduct screening tests Failure of medical practitioners to inform patients Financial constraints for patients who pay cash
**Munthali, A C et al., 2015, Malawi **[20]	Exploring barriers to the delivery of cervical cancer screening and early treatment services in Malawi: Some views from service providers	**Research method:** Qualitative **Data collection:** In-depth interviews	Not indicated n = 53	Lack of knowledge about cervical cancer among the general population Long distances to health facilities Services not offered on a daily basis Lack of spousal involvement Misconceptions about cervical cancer Gross shortage of staff Lack of equipment and supplies Lack of supportive supervision Gender and age of service providers
**Oketch, S Y et al., 2019, Kenya **[21]	Perspectives of women participating in a cervical cancer screening campaign with community-based HPV self-sampling in rural western Kenya: a qualitative study	**Research method:** Qualitative **Data collection:** In-depth interviews	Purposive n = 120	Social stigma associated with cervical cancer Long distance to screening sites (travel costs and travel time) Fear of pain during screening Embarrassment if male providers provided screening Fear of disease and death
**Mwaka, A D et al., 2013, Uganda **[22]	Mind the gaps: a qualitative study of perceptions of healthcare professionals on challenges and proposed remedies for cervical cancer help-seeking in post conflict northern Uganda	**Research method:** Qualitative **Data collection:** Key informant interviews (KIIs)	Purposive n = 15	Lack of awareness and knowledge about cervical cancer and service locations Lack of knowledge about the benefits of screening Lack of accurate knowledge of cervical cancer Financial constraints (screening costs) Discomfort with exposure of women’s genitals Perceived pain during pelvic examinations Lack of spousal support (emotional & financial) Few health facilities that provide screening Long distances to screening centers Lack of transport to screening centers Gender and age of service provider
**Ndejjo, R et al., 2017, Uganda **[23]	Knowledge, facilitators and barriers to cervical cancer screening among women in Uganda: a qualitative study	**Research method:** Qualitative **Data collection:** FGDs KIIs	Multistage (Random selection of sub counties and purposive selection of villages and participants) 10 FGDs (n = 119) KII (n = 11)	Lack of knowledge about cervical cancer and screening Lack of awareness about screening services availability Lack of facilities offering screening- services far away from the community Negative staff attitudes Staff shortages Lack of proper training to conduct screening Lack of screening materials Fear of discomfort during screening Gender and age of service provider Fear of a positive diagnosis Fear of finding out HIV status if provided with screening Financial constraints (transport, screening and treatment costs if found positive)
**Modibbo, FI et al., 2016, Nigeria **[24]	Qualitative study of barriers to cervical cancer screening among Nigerian women	**Research method:** Qualitative **Data collection:** FGDs	Purposive 4 FGDs (n = 49)	Lack of awareness of screening programmes Modesty concerns Gender of health care provider Fear of a positive result and disclosure of the results Fear of contacting other illnesses in the hospitals Discomfort during the screening process Denial of disease condition Discrimination (Islam women from their mode of dressing) Lack of husband’s permission for screening
**Fort, VK et al., 2011, Malawi **[25]	Barriers to cervical cancer screening in Mulanje, Malawi: a qualitative study	**Research method:** Qualitative **Data collection:** Interviews	Systematic n = 20	Low knowledge about cervical cancer and screening Misconceptions about screening (pulling out uterus) Fatalistic view of cervical cancer (fear of being diagnosed and dying soon) Low perceived risk Lack of time (too busy with household chores) Difficulty in navigating health care facilities Financial constraints (transportation and time) Long waiting times Lack of understanding on benefits of screening
**Nwankwo, K C et al., 2011, Nigeria **[28]	Knowledge attitudes and practices of cervical cancer screening among urban and rural Nigerian women: a call for education and mass screening	**Research method:** Quantitative **Data collection:** Interviewer- administered questionnaire	Convenience n = 1000	Lack of knowledge about cervical cancer screening No complaint Cannot afford the cost Cannot locate screening facility Screening is unnecessary Fear of a cancer diagnosis Never thought about it
**Compaore, S et al., 2016, Burkina Faso **[29]	Barriers to Cervical Cancer Screening in Burkina Faso: Needs for Patient and Professional Education	**Research method:** Quantitative **Data collection:** Interviewer- administered questionnaire	Convenience n = 351	Lack of awareness about cervical cancer and screening Low risk perception Not knowing where to go for screening Fear of being diagnosed with cervical cancer Long distance to screening site Financial constraints
**Tarwireyi, F., 2005, Zimbabwe **[30]	Perceptions and barriers to cervical cancer screening in a rural district of Mutoko, Mashonaland East Province, Zimbabwe	**Research method:** Quantitative **Data collection:** Interviews –assumption is that this was an interviewer-administered questionnaire	Multi-stage random n = 1 600	Lack of screening services at the nearest health centers Not yet ready for screening Lack of time Financial constraints – high transport costs Lack of knowledge of where to go for screening Long distance to health center Lack of proper policy to guide cervical cancer screening
**Kress, C M et al., 2015, Ethiopia **[31]	Knowledge, attitudes, and practices regarding cervical cancer and screening among Ethiopian health care workers	**Research method:** Quantitative **Data collection:** Self-administered multiple choice surveys	Purposive n = 335	Lack of necessary training to screen Lack of equipment and supplies for screening Lack of laboratory resources Screening tests too expensive to patients Difficulty to follow up with patients after screening
**Abiodun, OA et al., 2013, Nigeria **[40]	The understanding and perception of service providers about the community-based cervical screening in Nigeria	**Research method:** Quantitative **Data collection:** Self- administered questionnaire	Purposive n = 100	Low patient turnout due to a generally low level of awareness of cervical cancer and screening among the populace Lack of clear and comprehensive national cervical cancer management guidelines and policies in the region Lack of sustainability of screening service due to staff turnover Shortage of funds Inadequate consumables Shortage of skilled personnel Absence of budgetary allocation for cervical screening Lack of commitment by health personnel due to poor motivation Lack of hospital management and government support: women fail to get the service
**Okunowo, AA et al., 2018, Nigeria **[33]	Women's knowledge of cervical cancer and uptake of Pap smear testing and the factors influencing it in a Nigerian tertiary hospital	**Research method:** Quantitative **Data collection:** self-administered structured questionnaire	Convenience n = 144	Poor knowledge of cervical cancer My doctor has never advised me to do the test Poor knowledge about screening Low risk perception Lack of knowledge of where the test is done
**Perng, P et al., 2013, Tanzania **[34]	Promoters of and barriers to cervical cancer screening in a rural setting in Tanzania	**Research method:** Quantitative **Data collection:** Interviewer- administered questionnaire	Convenience quota sampling n = 300	Financial constraints (when cost barriers are removed, women who are less able to afford health care are more likely to participate) Perceived absence of ill health Age (younger and older women least likely to screen) Illiteracy
**Ebu, N I et al., 2015, Ghana **[35]	Knowledge, practice, and barriers toward cervical cancer screening in Elmina, southern Ghana	**Research method:** Quantitative **Data collection:** Structured interview schedule	Multistage random n = 392	Lack of screening sites Screening sites too far away Limited information on cervical cancer Absence of health education programmes Lack of adequate knowledge about the screening test and where it can be done Screening test is embarrassing and painful Religious values and cultural beliefs Lack of spousal support Low risk perception Fear of a cancer diagnosis and treatment Financial constraints (cost of the test unaffordable)
**Rosser, J I et al., 2015, Kenya **[36]	Barriers to Cervical Cancer Screening in Rural Kenya: Perspectives from a Provider Survey	**Research method:** Quantitative **Data collection:** Self-administered survey	Purposive n = 106	Staff shortages Lack of trained staff Insufficient space Insufficient supplies Inadequate knowledge of cervical cancer Long waiting times Gender of service provider Fear of pain with the speculum exam
**Chigbu, C O & Aniebue, U., 2011, Nigeria **[37]	Why southeastern Nigerian women who are aware of cervical cancer screening do not go for cervical cancer screening	**Research method:** Quantitative **Data collection:** Interviewer- administered questionnaire	Systematic sampling n = 3 712	Lack of adequate information Absence of symptoms Fear of violation of privacy Fear of outcome of results No family history of cervical cancer Distance to screening centers
**Titiloye, M A et al., 2017, Nigeria **[38]	Barriers to utilization of cervical cancer screening services among women of reproductive age in Ondo, Southwest Nigeria	**Research method:** Quantitative **Data collection:** Interviewer- administered questionnaire	Multi-stage n = 244	Fear of result Negative attitudes of health workers Husband’s influence on decision Screening procedure is painful Financial constraints (too expensive) Screening test not readily available Lack of knowledge on what age it is appropriate to go for screening Long distance to health facility Lack of time to get screened because it takes much time Health facility screening operational times not convenient Misconceptions about screening
**Abiodun, OA., et al. 2013, Nigeria **[32]	An assessment of women’s awareness and knowledge about cervical cancer and screening in Ogun State, Nigeria	**Research method:** Quantitative **Data collection:** Interviewer- administered questionnaire	Multi-stage random n = 2 000	Lack of awareness and knowledge on cervical cancer Lack of awareness and knowledge on cervical cancer screening Lack of interest Lack of access to screening
**Ibekwe, CM et al., 2011, Botswana **[39]	Perceived barriers of cervical cancer screening among women attending Mahalapye hospital, Botswana	**Research method:** Quantitative **Data collection:** Self-administered questionnaire for those who could read and write Interviewer-administered questionnaire for those who could neither read nor write	Convenience n = 300	Lack of information about the benefits of screening Low risk perception for cervical cancer
**Mangoma, J F et al., 2006, Zimbabwe **[26]	An assessment of rural women's knowledge, constraints and perceptions on cervical cancer screening: the case of two districts in Zimbabwe	**Research method:** Mixed—Quantitative and Qualitative **Data collection:** Quantitative Interviewer administered questionnaire Qualitative Semi-structured questionnaires to nurses & nurse aides In-depth interviews with health personnel FGDs with women Document analysis Narratives from 2 women suffering from cervical cancer & 1 who had hysterectomy	Quantitative Cluster random n = 356 Qualitative Purposive n = 29 n = 16 20 FGDs Hospital and clinical records n = 3	Lack of knowledge about the need for and importance of screening Lack of awareness about the local screening programme Gender of service provider Discomfort during screening procedure (lying on one’s back with legs open) Low level of knowledge and understanding about cervical cancer Absence of signs and symptoms Lack of money Men not understanding the importance of screening Absence of a screening programme Long distances to nearest screening sites Lack of trained nurses Lack of follow up (women referred for screening do not go because of lack of money, time and not understanding the consequences of the disease) Competing priorities (bread and butter issues) Misconceptions about cervical cancer (caused by witchcraft)
**Ngugi, C W et al., 2012, Kenya **[27]	Factors affecting uptake of cervical cancer early detection measures among women in Thika, Kenya	**Research method:** Mixed—Quantitative and Qualitative **Data collection:** Quantitative Interviewer- administered questionnaire Qualitative In-depth interviews	Not explained Quantitative n = 498 Qualitative n = 50	Lack of knowledge and awareness of cervical cancer and the benefits of screening Screening sites too far away Financial constraints (screening, treatment and transport costs) Fear of pain during the procedure Responsibility in the home (too busy with other household work and time spent at hospital is too long) Lack of spousal support Health workers not supportive of the programme (too busy even if women ask to be screened, no explanation of procedure before the test, rude to patients) Gender of service provider
**Getachew, S et al., 2019, Ethiopia **[41]	Cervical cancer screening knowledge and barriers among women in Addis Ababa, Ethiopia	**Research method:** Mixed—Quantitative and Qualitative **Data collection:** Quantitative Interviewer- administered questionnaire Qualitative FGDs	Quantitative Multi-stage n = 520 Qualitative Purposive 4 FGDs (n = 37)	Lack of symptoms Lack of knowledge regarding cervical cancer Lack of adequate information about the existence of screening, who is eligible for screening, where and when they should be screened Lack of screening services at the nearest health centers Health professionals do not promote screening

## Results

### Study characteristics

The key characteristics and findings of the 24 included articles are summarised in Table 3. The studies were published between 2005 and 2019. Eight were conducted in Nigeria, three in Kenya, two each in Uganda, Ethiopia, Malawi and Zimbabwe and one each in South Africa, Burkina Faso, Tanzania, Ghana and Botswana. Eight (33.3%) studies were qualitative, thirteen (54.2%) quantitative and three (12.5%) used the mixed method approach. Sixteen (66.7%) studies evaluated barriers to cervical cancer screening from the perspective of women who are the recipients of screening and six (25%) from the perspective of health service providers. Two (8.3%) evaluated the barriers from the perspective of both women and health service providers.

#### Qualitative studies

Purposive sampling was used in the majority of qualitative studies (6/8, 75%). For data collection, two studies each used In-depth interviews (25%) and FGDs (25%) respectively. The remaining four each used KIIs (12.5%), interviews (12.5%), a combination of FGDs and KIIs (12.5%) and a combination of interviews and field notes (12.5) respectively.

#### Quantitative studies

Of the 13 quantitative studies, 5 (38.5%) used convenience sampling. Multi-stage random sampling was used in 4 (30.8%), purposive sampling in 3 (23%) and systematic sampling in 1 (7.7%) study. Interviewer administered questionnaires were used for data collection in 8 (61.5%) studies and self-administered questionnaires in four (30.8%). One (7.7%) study used both self and interviewer-administered questionnaires depending on whether the participant could read and write. The sample size of the studies ranged from 100 to 3 712 participants.

#### Mixed methods studies

All three studies which employed both the qualitative and quantitative approaches used the interviewer-administered questionnaire for the collection of quantitative data. For the qualitative component, in-depth interviews and FGDs were each used in two studies, respectively. The third study used document analysis, FGDs, in-depth interviews and narratives from two women with a diagnosis of cervical cancer and one who had hysterectomy done. Findings from the narratives were not used in this systematic review as they were obtained from participants who did not meet the eligibility criteria for inclusion.

### Barriers to cervical cancer screening

Overall, 28 screening barriers were identified from the perspectives of service recipients, and 10 from the perspectives of service providers. Mostly cited by women were; inaccessibility of screening services, lack of awareness and knowledge on cervical cancer and screening benefits, and financial and socio-cultural constraints. Service providers perceived lack of training necessary to conduct screening, lack of equipment and supplies, staff shortages and gender and age of the health practitioner as major barriers to screening provision. Thematic analysis based on the socio-ecological framework which grounded the review yielded five a priori themes namely: health-system related, individual level, interpersonal, community related, and structural barriers. All the themes were not country-centric and could be transferrable between geographical settings in the region.

#### Health system related barriers to cervical cancer screening

##### Inaccessibility of screening services

Lack of access to screening services was identified as the key barrier to screening. Women maintained that screening services were not available at their local health facilities [23, 26, 27, 30, 32, 35, 38, 41]. The long distances they had to travel to reach the nearest screening sites usually located at tertiary levels of health care, were a deterrent to screening [18, 21, 26, 29, 30, 35, 37, 38]. This also has financial implications in terms of transport costs and lost time. Screening facilities’ operational times not amenable with women's schedules also posed a challenge and limited their chances of screening [38]. Those who had physical access to screening facilities found it difficult to navigate their way to the right place as information and directions were in most cases not readily available [25].

Service providers concurred that health facilities that provide screening were few [22] and far away from communities [23]. This resulted in women having to travel long distances to get screened, while not all facilities offered the service on a daily basis [20]. Transport to get to screening centers was also a challenge [22]. Access to screening is thus affected by unavailability of local screening facilities, transport constraints and screening operating times which are not user-sensitive.

##### Limited funding for cervical cancer programmes

Lack of a dedicated budget for cervical cancer programmes was highlighted as a barrier as it resulted in insufficient resources required to provide screening [40]. This included space for the provision of efficient screening services, [20, 36] and technical support to monitor the programme and provide guidance to service providers [20, 40]. Follow-up of patients who required further management also posed a challenge for health personnel, thus defeating the whole purpose of screening [26, 31].

##### Lack of skilled providers

Service providers maintained that shortage of personnel is a major hindrance to the uptake of screening considering that staff well equipped in the provision of the service is in short supply [20, 23, 36, 40]. Consequently the available trained personnel are not able to meet the demand [20, 26, 36]. This is also attributable to the high staff turnover among the trained cadres, [40] and lack of training opportunities for the available nurses and doctors [31]. Furthermore, trained providers are assigned to areas not related to screening, thus negatively affecting the availability of screening services [20, 40]. At some health facilities, the same personnel who provided screening were also responsible for rendering other maternal and child health services, which increases the workload and reduces their motivation [20]. Accordingly, the time within which screening sites are operational is limited due to the multiplicity of tasks skilled staff have to perform.

##### Lack of equipment and supplies

A general shortage of equipment and screening consumables was identified by service providers as a barrier to screening [20, 23, 30, 31, 36, 40]. Facilities often run out of supplies and cryotherapy is sometimes not provided due to broken down equipment which cannot be repaired for lack of funds [20].

##### Negative attitudes of service providers

Four studies; three [23, 27, 38] from the perspective of women and one [40] from the perspective of service providers highlighted negative attitudes of health personnel as an important reason for women's failure to seek screening. Women report that health workers are uncooperative and hostile to them. Such inappropriate behaviour leaves them with no option but to consult traditional healers for health care [23]. When women request screening, health workers allege to be too busy, and if the service is provided, no explanation related to the procedure is given [27]. Consistent with this, service providers argue that due to poor motivation, they lack commitment to efficiently provide the service. Such behaviours deprive women access to the screening services which they require [40].

#### Individual level barriers to cervical cancer screening

##### Lack of access to screening information

Women generally lack awareness of cervical cancer as a disease of public health concern [18, 29]. Those who may have heard about the disease have no full knowledge of its risk factors, prevention, and signs and symptoms [23, 25–27, 32, 33, 35, 41]. In concurrence, service providers attribute the low screening uptake to women's low levels of awareness about cervical cancer [20, 22, 40]. This consequently does not give women the motivation to seek screening. Moreover, women often have inadequate [36] and inaccurate [22] knowledge on cervical cancer and screening [23–25, 29, 32, 33, 37]. Regrettably, some women lack information on the existence of screening programmes even where such services are available locally [26], are not aware of the location of screening sites [18, 23, 28–30, 33, 35, 41], the appropriate age for screening [38, 41], and the need and benefits of screening [26, 27]. This dearth of information is partly due to poor information dissemination by health workers as indicated by both service recipients [18, 33, 41] and service providers including private practitioners [19], and absence of relevant health educational programmes [35]. In addition, service providers have highlighted that health professionals especially at the lower levels of care lack adequate knowledge on cervical cancer and its prevention and control and are therefore not able to give women up to date screening related information [22].

##### Financial constraints

Lack of financial resources was reported as a common obstacle to participation in cervical cancer screening. The cost of the test was considered as expensive by some women [23, 27, 28, 35] and service providers [19, 22, 31]. This is partly linked to the hidden costs associated with screening since the service is offered for free in most public health facilities. The indirect costs include high transport charges to screening sites [21, 23, 25, 27, 30], time lost on travel, [21, 25] long waiting times before screening [25, 30, 36] which could have been used productively, and lack of money to pay for treatment should the screen test yield a positive result [22].

##### Attitude of indifference to screening

The perception that screening is unnecessary [28] and not important [18] was noted as an impediment to screening. Women see no benefit in early detection measures as they believe that one would not be cured anyway, and still die of cancer [27]. Women also suggested that they had never thought about screening [28] and therefore were not ready for the test [30], or had no interest in getting screened [32]. These negative attitudes could be emanating from their lack of symptoms [26, 28, 34, 37, 41] which instils a notion of good health and therefore finding no reason to get screened. Women also believed they were not at risk for cervical cancer [18, 25, 33, 39], while some were not aware of their being at risk for the disease [29] and therefore felt no need for screening.

##### Fear of procedure and outcome

Fear of pain during the procedure was identified as a screening deterrent [23, 24, 35, 38]. Women receive negative information from friends [27], or have themselves had bad screening experiences and therefore avoid repeat screens [23]. Service providers also reported that women are not comfortable with pelvic examinations and fear that insertion of the speculum causes pain, hence will not participate in screening [22, 36]. For some women, fear of the possibility of receiving a positive result was a barrier [18, 21, 23–25, 29, 35, 37, 38]. Finding bliss in ignorance was associated with; fear of being left by spouse if known to have cervical cancer as that was thought to interfere with sexual relations [23], fatalistic view of cervical cancer, therefore finding it better not to know [18, 21, 24, 29, 37], fear of disclosure of results which may result in stigmatisation [24], fear of the side effects of treatment [35] and worry which may lead to an early death [38]. Women also expressed fear of contracting other diseases from the screening equipment and finding out their Human immunodeficiency virus (HIV) status if cervical cancer screening was linked to HIV screening [23].

#### Interpersonal barriers to cervical cancer screening

##### Lack of spousal support

Spousal or male partner support was found to be an important factor in the practice of screening because of the patriarchal nature of the African society. Husbands were revealed to have an influence on the decision for screening [38]. Women require their husbands’ permission to get screened for financial and cultural reasons [24] and since some men do not understand the importance of screening [26], they refuse to give their consent [35]. Women get accused of being promiscuous if they express their wish to screen because of the association of cervical cancer with a sexually transmitted virus [27]. Men can thus be a hindrance to screening. Service providers confirmed men’s lack of emotional and financial support for screening [22] expressed by women [26, 27, 35, 38]. Lack of male support for screening also creates barriers to treatment adherence if the woman has a positive result [20].

##### Misconceptions about cervical cancer

Negative connotations linked to cervical cancer and screening within women’s social circles has been identified as a big barrier to screening. The misconception that cervical cancer is associated with promiscuity deters women from screening as they do not want to be labelled as being promiscuous [38]. Additionally, women are misinformed and made to believe that use of the speculum during the test enlarges the vagina [20], that the uterus is pulled out for examination and reinserted after screening [20, 25], and that they may not be able to have children after screening. Subsequently, they get discouraged from utilising the service. Women’s screening behaviour is thus often subject to the influence of family and friends.

#### Community-related barriers to cervical cancer screening

##### Family responsibilities

Six studies revealed that women lacked time to attend screening due to family responsibilities. As household managers whom society expects to place the wellbeing of the family before their own, women have many competing priorities related to family survival which deprive them of time for screening [18, 26]. They are too busy with household chores to go to health facilities for preventive health services [25, 27, 30] relative to curative care. Some have no household help and find it hard to leave their tasks unattended since the time it takes to complete the screening processes is long [27, 38].

##### Socio-cultural and religious beliefs

It is very difficult to clearly distinguish between cultural and religious considerations as these two are intricately related. The same factors reported by some women as religious were reported as cultural by others. Consequently, socio-cultural and religious beliefs and gender and age of service provider cannot be discussed independently of each other.

Some women consider participation in cervical cancer screening as inappropriate and against their cultural and religious beliefs [35]. African women are generally conservative and suffer embarrassment at lying on their backs with their legs open [22, 26] and exposing their private parts for examination [22, 27, 35], especially if it is a male providing the service [21]. Exposure of genitals is viewed as a violation of women’s privacy [37]. The cultural and religious norms which some women value discourage them from exposing their intimate body parts to other people other than their husbands, unless if there are compelling reasons [24]. Modesty, embarrassment and religious beliefs are thus significant barriers to the utilisation of screening services.

Gender and age of the service provider were seen to pose a cultural barrier to participation in cervical cancer screening programmes. Women feel ashamed, shy, embarrassed, anxious and uncomfortable if males provide the service [23, 24, 26, 27]. Service providers echoed that gender of the provider interfered with screening programmes as women do not like their private parts exposed to male practitioners particularly if they have no gynaecological problems [19, 20, 22, 36]. Furthermore, older women are not willing to be screened by younger male health workers who they consider as their sons [19, 20, 22, 23, 26]. This is attributed to cultural norms. The same sentiments in relation to gender and age of service provider are obtaining in the private sector [19].

##### Social stigma associated with cervical cancer and screening

One study revealed that women decline cervical cancer screening because of the stigma associated with having cervical cancer [21]. They avoid going to screening sites because people may think they have the disease and suffer societal rejection. Stigma related to misconceptions was again mentioned by service providers as one of the perceived patient factors that inhibit screening uptake [36].

#### Structural barriers

Over and above the health system, individual, interpersonal and community related barriers to screening, clear comprehensive cervical cancer management policies and guidelines to guide cervical cancer screening and systematic cervical cancer screening programmes are not readily available in the region [26, 30, 40]. Efforts to prevent cervical cancer are therefore haphazard, and this has a negative impact on screening [26]. Where available, the policies are weak and characterised by a lack of political will and backing by governments. Inadequate funding of the programme results in poor availability of all resources necessary for screening due to the low priority which cervical cancer screening is given within the health system [40].

### Suggested strategies for addressing barriers to cervical cancer screening

Women and health service providers mutually suggest that; increasing access to cervical cancer screening within communities by addressing transport challenges [23], creating and raising awareness on screening through community mobilisation and sensitisation [20, 23, 26], assigning female staff to conduct screening [23], availing more skilled staff and supplies for the screening programme, and a collaborative approach at crafting policies that accord screening priority like other maternal and child health programmes [35], would improve the uptake of screening.

## Discussion

This systematic review synthesised findings of the key barriers to the uptake of cervical cancer screening from 24 studies conducted in 11 African countries. The barriers were presented from the viewpoint of service recipients and providers. Our analysis across the included studies indicate lack of information on the importance of screening and poor access to screening services as the most predominant barriers to cervical cancer screening in the region. Concordance of themes was demonstrated between qualitative and quantitative studies, and between women and service providers’ perspectives. Triangulation of findings was thus achieved [42].

Based on the findings of this review, factors that negatively impact cervical cancer screening in Africa are multidimensional and although common between countries, vary in magnitude from one setting to another. At the level of the health system, restricted access to screening in particular; lack of local health facilities that provide screening services, and prohibitive distances and cost to screening sites were shown to be the biggest challenges in the uptake of screening. The findings compare well with other studies conducted among indigenous populations worldwide [7], in Sub-Saharan Africa [10], in the Pacific [43] and in other middle and upper- middle income countries such as Turkey, Thailand, Jamaica and China which also report poor access to screening services due to various structural and health system related factors[44].

Most reviewed studies have advanced relatively similar recommendations for addressing the barriers to cervical cancer screening at different time periods yet, the uptake of screening has only slightly improved overtime. Our study attributes this to the fragmented tackling of the socio-ecological framework linked barriers independently of each other, and postulates that responding to challenges at only one level of the framework has the effect of increasing the barriers at a different ignored level. For example; increasing awareness and knowledge on screening among women has the likelihood of increasing the demand for the service. However, if screening facilities are not concurrently increased, the challenge will shift from the demand to the supply side. Our review further hypothesises that even if all other barriers could be addressed, screening incidence would still remain low if screening facilities are not universally rolled out to communities. Evidenced to this is the effective screening programmes in high-income countries that have resulted in low cervical cancer incidence [45]. Access to services is central to screening uptake in view of the fact that women cannot engage in cervical cancer screening if there are no services to deliver it [43]. This therefore requires the development of context specific innovative policies and strategies, or the modification of existing ones to make the service readily accessible to all women who need it.

One long term solution which has been recommended in previous studies but has not been universally applied is the inclusion of cervical cancer prevention and control into the nurses’ pre-service training curriculum [43, 46]. Our study further recommends that the cervical cancer component be examinable both theoretically and practically to ensure nurses would have acquired the necessary skills upon completion of their training. This is because nurses constitute the most authoritative source of health information especially for women [47] and are available at all levels of health care. To address the associated financial constraints which have been raised as prohibitive to this recommendation, the training could make use of the already existing resources since all teaching hospitals are likely to have screening units. Screening coverage would consequently be ensured at all levels of health care given the availability of other necessary resources, which may however not be readily available in all settings in the short term. However, women would still benefit from receiving accurate information on cervical cancer prevention and control to enable them to seek screening services where available,

The World Health Organization has also provided guidelines on the attainment of universal screening coverage, its scalability and sustainability [48], which African countries need to modify and implement. Furthermore, the World Health Organization states that the success of the drive to eliminate cervical cancer depends on political will and country-led action investments [49]. This is particularly required in African countries for the economic support of cervical cancer screening programmes and development or more effective implementation of country-centric policies and guidelines for screening. Nevertheless, individual and interpersonal factors within the socio-ecological model still need to be addressed given that some low resource countries in Africa with a strong political will still report low screening rates [50]. Considering alternative screening delivery models like mobile clinics is another viable option that has proved to be effective in other low resource settings [46]. This should be strengthened or implemented in settings that have not introduced it.

Lack of awareness and knowledge about cervical cancer and screening was commonly reported in this review although not identified as the primary barrier to screening, contrary to findings from previously conducted reviews [9, 10, 43, 44, 51, 52]. This variance could be a result of on-going awareness campaigns and improved education of women about the disease and its prevention, which could be an indication that knowledge about cervical cancer and screening is progressively improving. The limited knowledge that women have on cervical cancer and screening has been linked to failure by health professionals to educate their communities appropriately. A number of studies conducted in similar settings have reported similar findings [9, 11, 43]. Effective health education is likely to improve women’s knowledge about the disease and enhance the uptake of screening [15, 33]. Facilitation of intrinsic motivation through establishing systems for continuing knowledge and skills training of health professionals in cervical cancer prevention could help in the scale up of screening coverage to address this gap. More opportunities for the education of communities including men need to be explored in a culturally competent manner using affordable and available resources. Community Health Workers for example, possess authority and influence and are respected in their communities. Such authoritative sources of cultural knowledge could be harnessed and trained to complement the efforts of health professionals in disseminating knowledge on cervical cancer screening. A clearer understanding and increased knowledge among women could dispel myths and misconceptions about cervical cancer and screening and could result in an increased demand for the service. For women to participate in screening, they need to have knowledge of the disease and how it is screened [43]. As stated by some women; “it is not possible to use what they don’t know about” [18].

Our review identified that at the interpersonal and community levels of the socio-ecological model, women are essentially constrained from screening by cultural and religious factors. This finding is consistent with other studies which confirm that women need to seek approval and funding from their spouses or partners to enable them to access cervical cancer screening [15]. Such approval is at times denied for varying reasons [11, 44, 52, 53]. Moreover, women may also be discreet in discussing reproductive health issues with their spouses for cultural reasons [46], while husbands are not expected to be involved in talking about women’s health issues [11, 54]. Male involvement in reproductive health services needs further support to enhance women's attendance for screening.

The provision of screening services by males has been seen to discourage women, particularly the older ones, from seeking screening. Findings of this review are congruent with evidence from other studies [15, 53, 55, 56]. For some cultures, it is taboo foe females to expose their nudity to males other than their sexual partners and is contradictory to their and values [10]. On the contrary, some studies conducted among minority groups in Canada revealed that women felt uncomfortable discussing or undertaking the screening test irrespective of the sex of the service provider [9]. The differences in findings could be related to cultural beliefs.

However, despite the religious taboos and social stigmas associated with screening, women still respect health providers’ opinions and recommendations [9]. This reiterates the critical role health workers have in educating women on cervical cancer and screening at every interaction with women for enhanced utilisation of screening services. Evidently, there is a need to change some socio-cultural beliefs if uptake of screening is to increase. This however is a challenging task since women’s understanding of issues is grounded on religious and cultural traditions and makes promotion of screening difficult to address in isolation to those traditions [52]. Accordingly, a simple educational intervention is unlikely to achieve the desired result. Rather than targeting just the women with cervical cancer screening messages, educational interventions should target all levels of the socio-ecological framework and be extended to include families, communities and traditional/religious leaders who could serve as change agents in support of promotive and preventive health programmes that include cervical cancer screening.

## Limitations

Although the search strategy was tailored for studies on barriers to cervical cancer screening conducted in Africa, this was not achievable as no articles were retrieved from North and Central African countries. Screening barriers unique to these countries were therefore not explored. Evidence suggests that cervical cancer is uncommon in Northern Africa [3] which could be the reason for lack of research in that area. Nevertheless, findings of this review exclude an important segment of the study population which could be having unique barriers to cervical cancer screening. In addition, grey literature which could have provided useful insights for the review was excluded. Despite these limitations, the overall findings were consistent across the studies and can be extrapolated to similar geographical settings in Africa.

## Conclusions

In this systematic review, we thematically explored the factors that prevent women from seeking cervical cancer screening services in Africa. Barriers to screening were found to be multi-dimensional spanning all levels of the socio-ecological framework. Poor access to screening facilities, lack of comprehensive knowledge on cervical cancer and screening, and socio-cultural influences were found to be the key factors that contribute to the sub-optimal uptake of cervical cancer screening among women in African countries. From the view of health personnel, trained service providers were insufficient to meet the demand for screening. Similarly, screening equipment is not adequate for the delivery of a comprehensive service.

While women could have the essential knowledge on cervical cancer and get the motivation for screening, geographical, social and financial inaccessibility of the service could prevent them from screening. Conversely, women with full understanding of screening benefits and easy access to screening may still fail to utilise the service if they find it unacceptable due to intrapersonal and community influences related to religion and culture, and health system factors. Our study elucidates the criticality of tackling the barriers to screening at all levels of the socio-ecological model in a structured manner that would prevent increasing barriers at another level in the process.

Success at achieving a high uptake of screening should therefore focus on concurrently addressing all screening barriers at the individual, interpersonal, community, health systems and structural levels and apply the primary health care model which supports the availability, accessibility, acceptability and affordability of services with full community involvement. Application of this holistic approach could provide solutions that are responsive to communities and health services’ needs. There is also a need for dedicated cervical cancer programmes budgets to make available all the required resources for screening. Our review provides insights into the need for long-term strategies to reduce screening barriers at all levels of the socio-ecological model based on the needs of the community for achieving and sustaining high screening rates. Further research is required to investigate the feasibility and cost effectiveness of this multifaceted approach.

### Supplementary Information

**Additional file 1. **

## Data Availability

All data generated or analysed during this study are included in this published article.
