# Supplementary material for: Barriers to cervical cancer screening in Africa: a systematic review
Source: BMC Public Health. 2024 Feb 20;24:525. doi: 10.1186/s12889-024-17842-1 (PMC10877795; doi:10.1186/s12889-024-17842-1)
Supplement: Supplementary file 1 — Additional file 1. [file 12889_2024_17842_MOESM1_ESM.docx]

- 1. **Supplementary file 1: Search histories**

**PubMed (Medline):**

| #1 | (cancer* or tumor* or tumour* or neoplas* or carcinoma* or adenocarcinoma* or malignan*) Field: Title/Abstract |
| --- | --- |
| #2 | Cervi* Field: Title/Abstract |
| #3 | #1 AND #2 |
| #4 | "Uterine Cervical Neoplasms"[Mesh] or "Uterine Cervical Dysplasia"[Mesh] |
| #5 | #3 OR #4 |
| #6 | "Vaginal Smears"[Mesh] |
| #7 | "Papanicolaou Test"[Mesh] |
| #8 | (vagina* or PAP or cervi*) AND (smear* OR test* OR screen* OR cytology or cytobrush) Field: Title/Abstract |
| #9 | "Acetic Acid"[Mesh] |
| #10 | “visual inspection” OR VIA Field: Title/Abstract |
| #11 | Cervicography Field: Title/Abstract |
| #12 | "Mass Screening"[Mesh] |
| #13 | (#6) OR #7 OR #8 OR #9 OR #10 OR #11 OR #12 |
| #14 | "Africa"[Mesh] |
| #15 | algeria OR angola OR benin OR botswana OR burkina faso OR burundi OR cameroon OR cape verde OR central african republic OR chad OR comoros OR congo OR "Democratic Republic of Congo" OR DRC OR djibouti OR equatorial guinea OR egypt OR eritrea OR ethiopia OR gabon OR gambia OR ghana OR guinea OR bissau OR ivory coast OR (Côte d' Ivoire) OR jamahiriya OR kenya OR lesotho OR liberia OR Libya OR madagascar OR malawi OR mali OR mauritania OR mauritius OR mayotte OR morocco OR mozambique OR namibia OR niger OR nigeria OR principe OR reunion OR rwanda OR "Sao Tome" OR senegal OR seychelles OR "Sierra Leone" OR somalia OR "South Africa" OR st helena OR sudan OR swaziland OR tanzania OR togo OR tunisia OR uganda OR zaire OR zambia OR zimbabwe OR "Central Africa" OR "West Africa" OR "East Africa" OR "Southern Africa" OR "South Africa" Field: Title/Abstract |
| #16 | Search (#14) or #15 |
| #17 | Search (#5) AND #13 AND #16 |

**Database: Embase 1947- May 2019:**

| 1 | ((cancer* or tumor* or tumour* or neoplas* or carcinoma* or adenocarcinoma* or malignan*) adj2 (cervix or cervical)). ab. or ((cancer* or tumor* or tumour* or neoplas* or carcinoma* or adenocarcinoma* or malignan*) adj2 (cervix or cervical)).ti. |
| --- | --- |
| 2 | uterine cervix cancer/ |
| 3 | uterine cervix dysplasia/ |
| 4 | 1 or 2 or 3 |
| 5 | Papanicolaou test/ or vagina cytology/ or vagina smear/ or uterine cervix cytology/ or vaginal smear*.mp. |
| 6 | ((vagina* or PAP or cervi*) adj2 (smear* or test* or screen* or cytology or cytobrush)). ab. or ((vagina* or PAP or cervi*) adj2 (smear* or test* or screen* or cytology or cytobrush)).ti. |
| 7 | acetic acid.mp. or acetic acid/ |
| 8 | "visual inspection".ab. |
| 9 | "visual inspection".ti. |
| 10 | cervicography.mp. or colposcopy/ |
| 11 | 5 or 6 or 7 or 8 or 9 or 10 |
| 12 | 4 and 11 |
| 13 | exp Africa/ or africa.mp. |
| 14 | ("Central Africa" or "West Africa" or "East Africa" or "Southern Africa").mp. |
| 15 | (algeria or angola or benin or botswana or burkina faso or burundi or cameroon or cape verde or central african republic or chad or comoros or congo or "Democratic Republic of Congo" or DRC or djibouti or equatorial guinea or egypt or eritrea or ethiopia or gabon or gambia or ghana or guinea or bissau or ivory coast or cote d ivoire or jamahiriya or kenya or lesotho or liberia or libya or madagascar or malawi or mali or mauritania or mauritius or mayotte or morocco or mozambique or namibia or niger or nigeria or principe or reunion or rwanda or "Sao Tome" or senegal or seychelles or "Sierra Leone" or somalia or "South Africa" or st helena or sudan or swaziland or tanzania or togo or tunisia or uganda or zaire or zambia or zimbabwe).mp. |
| 16 | 13 or 14 or 15 |
| 17 | 12 and 16 |

**Cinahl (EBSCOHost):**

| # | Query |
| --- | --- |
| S15 | S14 AND S14 |
| S14 | TX ( africa or african ) OR ( algeria or angola or benin or botswana or burkina faso or burundi or cameroon or cape verde or central african republic or chad or comoros or congo or "Democratic Republic of Congo" or DRC or djibouti or equatorial guinea or egypt or eritrea or ethiopia or gabon or gambia or ghana or guinea or bissau or ivory coast or cote d ivoire or jamahiriya or kenya or lesotho or liberia or libya or madagascar or malawi or mali or mauritania or mauritius or mayotte or morocco or mozambique or namibia or niger or nigeria or principe or reunion or rwanda or "Sao Tome" or senegal or seychelles or "Sierra Leone" or somalia or "South Africa" or st helena or sudan or swaziland or tanzania or togo or tunisia or uganda or zaire or zambia or zimbabwe ) |
| S13 | S7 AND S12 |
| S12 | S8 OR S9 OR S10 OR S11 |
| S11 | "papanicolaou test" |
| S10 | "cervicography" |
| S9 | "acetic acid OR visual inspection OR visual inspection with acetic acid" |
| S8 | TX (vagina* or PAP or cervi*) N2 (smear* or test* or screen* or cytology or cytobrush) |
| S6 | TX cervical cancer OR TX cervical neoplasm* OR TX cervical carcinoma* |
| S5 | S3 OR S4 |
| S4 | S1 OR S2 OR S3 |
| S3 | "(cancer* or tumor* or tumour* or neoplas* or carcinoma* or adenocarcinoma* or malignan*) N2 (cervix or cervical)" |
| S2 | (MH "Cervical Intraepithelial Neoplasia") |
| S1 | (MH "Cervix Neoplasms") |

**SCOPUS:**

( ( TITLE-ABS-KEY ( namibia OR niger OR nigeria OR principe OR reunion OR rwanda OR "Sao Tome" OR senegal OR seychelles OR "Sierra Leone" OR somalia OR "South Africa" OR "st helena" OR sudan OR swaziland OR tanzania OR togo OR tunisia OR uganda OR zaire OR zambia OR zimbabwe ) ) OR ( TITLE-ABS-KEY ( "guinea bissau" OR "ivory coast" OR "cote d ivoire" OR jamahiriya OR kenya OR lesotho OR liberia OR libya OR madagascar OR malawi OR mali OR mauritania OR mauritius OR mayotte OR morocco OR mozambique ) ) OR ( TITLE-ABS-KEY ( "cape verde" OR "central african republic" OR chad OR comoros OR congo OR "Democratic Republic of Congo" OR drc OR djibouti OR "equatorial guinea" OR egypt OR eritrea OR ethiopia OR gabon OR gambia OR ghana ) ) OR ( TITLE-ABS-KEY ( algeria OR angola OR benin OR botswana OR burkina AND faso OR burundi OR cameroon OR cape AND verde OR central AND african AND republic OR chad OR comoros OR congo OR "Democratic Republic of Congo" OR drc OR djibouti OR equatorial AND guinea OR egypt OR eritrea OR ethiopia ) ) OR ( TITLE-ABS-KEY ( africa* ) ) ) AND ( ( TITLE-ABS-KEY ( "cervical cancer*" OR "cervical neoplasm*" OR "cervical dysplasia" ) ) AND ( TITLE-ABS-KEY ( "PAP smear" OR "Papanicolau smear" OR "vaginal smear*" OR "acetic acid" OR "visual inspection" ) ) )
